# Visual Detection of Speckles in the Fish *Xenotoca variata* by the Predatory Snake *Thamnophis melanogaster* in Water of Different Turbidity

**DOI:** 10.1371/journal.pone.0129429

**Published:** 2015-06-10

**Authors:** Javier Manjarrez, Eric Rivas-González, Crystian S. Venegas-Barrera, Alejandro Moyaho

**Affiliations:** 1 Laboratorio de Biología Evolutiva, Facultad de Ciencias, Universidad Autónoma del Estado de México, Toluca, Estado de México, México; 2 División de Estudios de Posgrado e Investigación, Instituto Tecnológico de Ciudad Victoria, Ciudad Victoria, Tamaulipas. México; 3 Laboratorio de Ecología de la Conducta, Instituto de Fisiología, Benemérita Universidad Autónoma de Puebla, Puebla, México; University of Windsor, CANADA

## Abstract

Semi-aquatic snakes integrate visual and chemical stimuli, and prey detection and capture success are therefore linked to the display of visual predatory behavior. The snake *Thamnophis melanogaster* responds preferentially to individuals of the fish *Xenotoca variata* with a greater number of bright, colorful spots (lateral speckles) compared with those with a smaller number; however, water turbidity can reduce underwater visibility and effect the vulnerability of fish. In this study, we tested whether the presence of iridescent speckles on the flanks of male *X*. *variata* interacted with water turbidity to modify the predatory behavior displayed by the snake *T*. *melanogaster*. We predicted that in an experimental laboratory test, the snakes would increase the frequency of their predatory behavior to the extent that the water turbidity decreases. The snakes were tested at six different levels of water turbidity, in combination with three categories of male fish (with few, a median number of, or many speckles). The results showed that in a pool with high or zero turbidity, the number of speckles is not a determining factor in the deployment of the predatory behavior of the snake *T*. *melanogaster* toward *X*. *variata*. Our findings suggest that snakes can view the fish at intermediate percentages of turbidity, but the number of speckles in male *X*. *variata* is irrelevant as an interspecific visual signal in environments with insufficient luminosity. The successful capture of aquatic prey is influenced by integration between chemical and visual signals, according to environmental factors that may influence the recognition of individual traits.

## Introduction

In aquatic foragers that visually detect prey, water turbidity can affect predatory efficiency due to reduced underwater visibility [1] and modify prey vulnerability [2,3]. Suspended particles result in water turbidity by absorbing and scattering light rays entering the water and thereby reducing the difference in brightness between an object and the background of the environment [4,5]. Most of the antipredatory behavior strategies of fish become ineffective in turbid water as the predator–prey interaction distance shortens, and little change in antipredatory foraging behavior is therefore observed among aquatic species [6]. The distance of visual prey detection is of paramount importance in the success of aquatic prey capture by snakes [7].

Color patterns and brightness are distinctive features of some species of aquatic animals, and their variation and evolution are explained by a balance between sexual selection, primarily based on mate choice, and natural selection by predators [8]. For example, individual brightness and body color intensity are maintained by sexual selection but can increase the visual detection during predation [9]. Similarly, male fish (*Xenotoca variata*) exhibit bright and colorful spots (hereafter referred to as speckles) on their flanks that attract females but also attract predatory snakes such as *Thamnophis melanogaster*, which respond preferentially to fish with a large number of speckles regardless of water turbidity [3,10,11]. These findings suggest that the risk of predation by *T*. *melanogaster* has led to selection against fish speckles in *X*. *variata*. Aquatic snakes of the genus *Thamnophis* are predators that use visual and chemical stimuli, and prey detection and capture success in these snakes are therefore linked to the display of visual predatory behaviors, such as their orientation, approach, and attacks on prey [12], even toward artificial prey [7].

However, little is known about how water turbidity alters visual predatory behaviors. In this study, we tested whether the presence of iridescent speckles on the flanks of *X*. *variata* males modifies the predatory behavior displayed by *T*. *melanogaster* snakes in a context with turbid water. We predicted that in laboratory experimental tests, the snakes would decrease the frequency of their visual predatory behavior as a function of increments in water turbidity.

*X*. *variata* is a viviparous fish (Subfamily Goodeinae) that shows strong sexual dimorphism in morphological and behavioral traits. Adult males have shiny lateral speckles, whose number varies between and within populations [3]. The snake *T*. *melanogaster* is endemic to the highlands of central Mexico [13]. It is considered a specialist aquatic species in its diet and foraging technique underwater, though it feeds on fishes, leeches, and tadpoles [12,14,15].

## Materials and Methods

### Ethics statement

The collection and manipulation of fishes and snakes were conducted with permits received for field work from the Mexican government through to the Ministry of the Environment and Natural Resources (SEMARNAT; permit SGPA/DGVS/07164). This study received the approval of the ethics committee of the Universidad Autonoma del Estado de Mexico.

### Experimental fish

We used 52 male *X*. *variata* fish from Lake Yuriria, Guanajuato (20°15´ N-101°7´ W) and three fish from Lake Cuitzeo, Michoacán (19°56´ N—101°6´ W). In the laboratory, we placed the fish in 40 L tanks with gravel as a substrate and an air pump, filters, and automatic thermostats to maintain the water temperature (20–27°C). The fish were fed commercially available dry food. The fish exhibited an average standard length of 4.27 ± 0.75 cm (mean ± ds) and 19.20 ± 14.51 speckles and were classified into three categories according to the number of speckles (Table 1). The standard length of the fish was related to the number of speckles (r2 = 13.5, p = 0.007), although the coefficient of determination explained a modest proportion of the variance. The fish were randomly chosen for the experiment, ensuring that they were smaller than the size of the snake’s mouth, and they were tested once a week to prevent mortality due to continuous exposure to suspended solids.

**Table 1:** Lateral speckles categories in male fish *X*. *variata*.

Speckles categories	Number speckles
**Many**	71–100
**Median**	39–70
**Few**	7–38

During the behavioral trial, the fish were always isolated physically from the snakes (as described below in the experimental tank section). They apparently ignored the presence of the snakes and swam continuously inside the compartment.

### Experimental snakes

We collected and used 13 adult male and 17 female *T*. *melanogaster* snakes (mean SVL 42.37 ± 10.06, range 34–66 cm) collected at the same localities from which the fish were captured (24 from Lake Cuitzeo and 6 from Lake Yuriria). In the laboratory, we measured, weighed, and sexed the snakes, after which the snakes were individually housed in tanks (51 × 26 × 28 cm) at a room temperature of 24.5–30°C; a lower temperature would impede their predatory activity [16]. The snakes were kept under a natural light–dark photoperiod and fed 12 hours after each experimental trial with a ration of live minnows and carp of approximately 2% of their body weight to stimulate their appetite during the trials.

### Experimental tank

**Fig 1:**
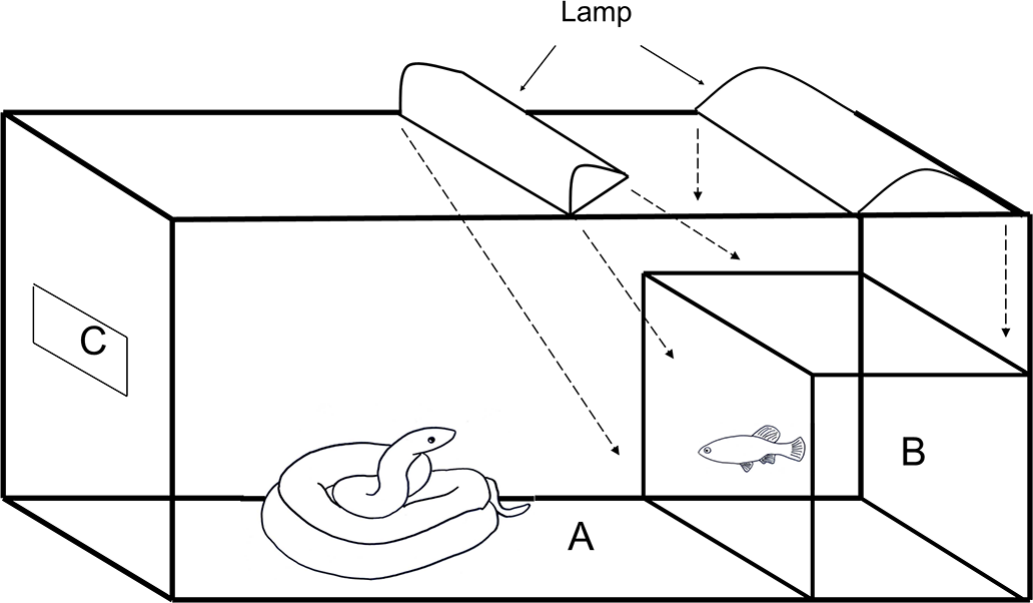
Experimental tank to follow the predatory behaviors displayed by snake *T*. *melanogaster* over the fish *X*. *variata*. A- compartment for experimental snake, B- pool filled with water containing sediments, into which the experimental fish were placed, C- window with dark blue cellophane paper to prevent the snakes from seeing the observer while he recorded the predatory behavior. The dashed arrows show the angle of incidence of two neon lamps light. One lamp was directed at an angle of 45° to the pool, and the other was above the pool so that light was reflected from the speckles of the male fish.

A 40 L glass tank (51 × 26 × 28 cm) was divided into two compartments: the compartment (28 × 39 × 26 cm) in which the experimental snake was maintained during the test was empty (Fig 1), and the other was filled with water containing sediments (20 × 26 × 12 cm), into which the experimental fish were placed (Fig 1). The dimensions of the pool allowed the snake to visualize the fish, which could even attempt to flee [17]. Between the two compartments, there was a barrier that prevented the snake from entering the pool. The tank was illuminated with two neon lamps of 15 watts each. One lamp was directed at an angle of 45° to the pool, and the other was above the pool so that light was reflected from the speckles of the male fish (Fig 1). The tank was covered with dark paper to block the passing of external visual stimuli. A small window was cut, and dark paper was replaced with dark blue cellophane paper to prevent the snakes from seeing the observer while he recorded the predatory behavior (Fig 1).

Sediments were suspended in the water to achieve the desired turbidity level, which was expressed as a percentage relative to the maximum concentration of suspended solids at which the fish can survive [18], as described below. A higher concentration of solids could increase the risk of fish mortality [18]. Sediments from Lake Yuriria were used to muddy the water. We did not use sediments from Cuitzeo because water pollution could harm the fish [19]. In the laboratory, the sediments were dried and sieved to obtain a concentration of 422 mg/l of suspended solids (particles of approximately 1 μ), which was considered 100% turbidity. From this concentration, we degraded turbidity to 80%, 60%, 40%, 20%, and 0% (Table 2). Water turbidity was measured using a spectrophotometer at a wavelength of 810 nm (HACH DR 5000), performing five measurements per sample. We did not employ any mechanism to maintain constant water turbidity, such as using an air diffuser, because we observed that bubbles altered fish behavior in pilot tests.

**Table 2:** Concentration of suspended solids (mg /l) in six concentrations of water turbidity.

Water turbidity (%)	Sediment (g/l)	Suspended solids (mg/l)^a^
100	3.33	422 ± 22.70
80	2.66	345.8 ± 27.57
60	2.00	249.6 ± 26.86
40	1.33	153 ± 26.81
20	0.66	83.6 ± 11.50
0	0	0

^a^Median ± SD

### Experimental design

Each snake was tested at each of six different percentages of water turbidity (100, 80, 60, 40, 20, and 0) and with each of three categories of male fish (with few, a median number of, or many speckles). We placed the snakes inside the experimental tank 24 hours before starting the trial. On the trial day, one male fish was placed in the pool at a water temperature of 18–22°C, which is an optimal temperature range for the predatory activity of *T*. *melanogaster* [16]. Then, 1 min later, we removed the barrier between the pool and the snake and began the trial.

### Behavioral trial

Each test lasted 30 min, or less if the snake attacked the fish. We observed and recorded the following predatory behaviors displayed by *T*. *melanogaster* [12]:

(1) Slow crawling—lateral undulation on the substrate; (2) orientation—displacement of the head toward the visual stimulus, which can be accompanied by rapid locomotion; (3) approach—slow or fast locomotion toward the prey; (4) attack—quick projection of the head of the snake toward the stimulus, either toward the front or side of the fish; (5) latency—which is considered a measure of motivation and defined as the time in which the snake exhibits the first deployment of predatory behavior (snakes that did not display predatory behavior were assigned a latency of 30 min); and (6) the distance of visual fixation—the distance between the glass separating the dry and water-containing compartments and the fish at the time when viewed by the snake. This distance was visually estimated as discrete units from 2 to 12 cm (for simplicity, this term is only referred to as "visual fixation"). When the fish is very close to the front glass of the experimental tank (distance from 0 to 2 cm), turbidity should not play a role at all, and these distances were not considered. Because of the brief duration of behaviors (1) to (4), we counted only the frequencies of the actions, regardless of their duration.

### Analysis

We determined whether there were significant differences in the six predatory behaviors displayed by the garter snake *T*. *melanogaster* depending the number of lateral iridescent specks on the male fish and water turbidity, for which we performed a linear discriminant function analysis (LDFA). LDFA is an inferential, descriptive multivariate procedure for testing differences between groups according to mean of all of the variables and for generating linear combinations that classify objects as a function of their characteristics [20], which is implemented in Statistica software (ver. 12. StatSoft 2012). The objective of LDFA was to test differences between groups and identify which variables discriminate between two or more groups. We compared 18 treatments (included in a categorical dependent variable), which were defined a priori and represented a combination of a gradient of the number of iridescent specks (few, median and many) and the percentage of turbidity (0, 20, 40, 60, 80, and 100%), in function of six predatory behaviors (continuous independent variable; S1 Table). LDFA reduces the dimensionality of the data through the generation of linear combinations of the original variables into a smaller number of variables that provides the most overall discrimination between groups (roots), where each new value (canonical scores) contains a fraction of the information from all of the original variables [21]. Thus, each observation exhibited a specific canonical value for each root, where observations with closer canonical values were associated with garter snakes with similar predatory behaviors, and distant values were associated with different predatory behaviors. The roots were orthogonal, that is, each root contributes a different amount of variation. The first root accounted for the largest amount of discrimination between groups, and subsequent roots explained less variation, which was not included in the preceding roots. The number of roots that were generated was equal to the number of groups minus one [22], and interpretation of the results was performed only using roots that contributed significantly (*X* ^2^ test with successive roots removed).

Comparisons between treatments were performed under the null hypothesis that the variations in the predatory behavior displayed by the snakes between treatments were similar to the behavioral variations deployed by the snakes within each treatment, and the estimated value was contrasted with the theoretical value of the *F*-distribution. We employed a probability of 0.05 to test the hypothesis, where *P* values lower than 0.05 were associated with groups of snakes showing different predatory behaviors, whereas values greater than or equal to 0.05 were associated with groups with similar predatory responses.

The canonical average of the observations from each group (centroid) for the significant roots was plotted, which reflects the general predatory behavior displayed by garter snakes given the number of iridescent specks and the percentage of turbidity. We do not plot all observations to ovoid saturate the graph, due the high number of treatments (18) and observations (326) and only we plot treatments centroids (S2 Table). The position of the centroids was interpreted using the variables that contributed most to discriminating between groups. We chose those variables that exhibited a coefficient of the factor structure higher than 0.5 or lower than -0.5. The coefficients represent the correlation between the original variables and the canonical scores (roots). Finally, we used classification function (S3 Table) to decide if the case belongs to the treatment for which it was classified (correct case classification) or present characteristics similar to another treatment (incorrect case classification; S4 Table). We reported the corrected classification percent of each treatment, which represent the percent of snakes that display more similar behaviors within treatment that between treatments. Higher corrected classification percent were associated to a high discrimination power, while small percent classification to lower discrimination power.

## Results

Water turbidity and the numbers of speckles affected the latency and visual fixation of the snakes (Wilks’ Lambda = 0.26; *F*_18, 540_ = 4.58; *P* < 0.0001). LDFA revealed that the first root explained 79.3% of the variation in the snakes’ predatory behavior (eigenvalue = 1.62). Visual fixation and orientation were the variables that contributed most to the differences between groups on the first root (Table 3). The second root explained 8.9% of the variation (eigenvalue = 0.20), which was mainly represented by differences in orientation and slow crawling between groups (Table 3). We found significant differences between groups, where the number of speckles did not affect the predatory behavior of the garter snakes under a higher (>60%) or lower percentage of turbidity (<20%), though they played a major role at intermediate percentages of turbidity (40–20%). At lower turbidity, the garter snakes could fix the fish at a greater distance and increase their orientation compared with higher turbidity, where the garter snakes increased their attack latency. At 20 and 40% turbidity, the garter snakes displayed greater orientation and slower crawling toward fishes with high number of speckles than fishes with intermediate and lower numbers.

**Table 3:** Factor structure of the first two roots for LDFA of each behavioral response displayed by snake *T*. *melanogaster* over male fish *X*. *variata*.

Variable	Root 1	Root 2	Mean ± DS
**Visual fixation**	0.8123	-0.0993	3.4 ± 1.2 cm
**Orientation**	0.6135	0.5639	2.6 ± 1.5
**Latency**	-0.3184	0.0421	10.2 ± 3.9 min
**Slow crawling**	0.1576	0.4977	3.5 ± 0.7
**Attack**	0.0784	0.3313	0.3 ± 0.1
**Approach**	0.4214	-0.0416	1.1 ± 0.7

**Fig 2:**
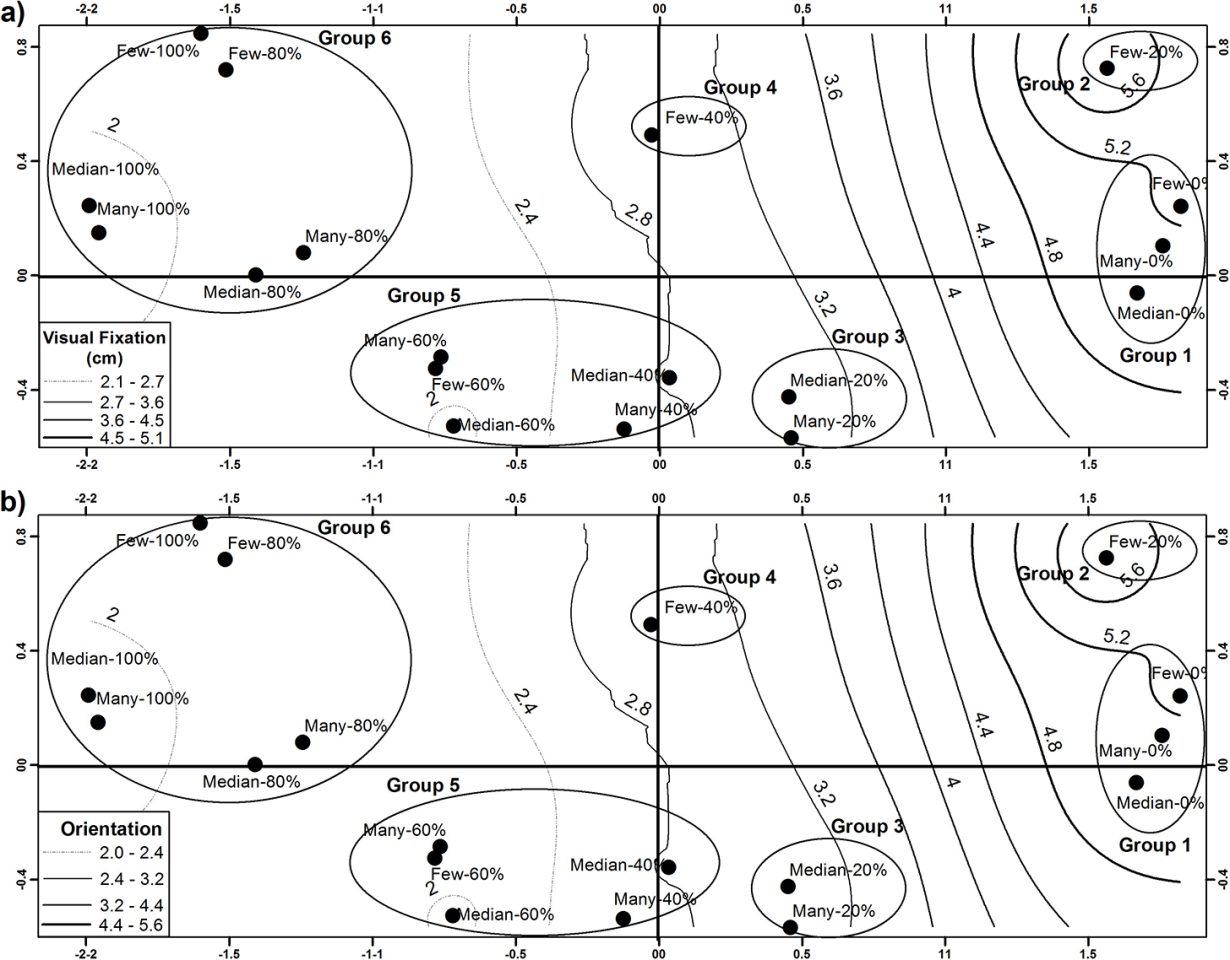
Plot showing average position (centroid) of predatory behavior of 18 treatments displayed by snake *T*. *melanogaster*, depending of three categories (few, median, many) of number of lateral iridescent specks, on the male fish *X*. *variata* and five categories of water turbidity (100, 80, 60, 40, 20 and 0%). The first two roots of LDFA account the 88.2% variability. The position of centroids on the first root were positively associated to variations to visual fixation (a) and orientations of snakes (b), while on the second root centroid position were positively associated to orientation and slow crawling. Plot show contours of visual fixation and orientation displayed of snakes when snakes were exposed to a gradient of turbidity and number of speckles.

Based on pairwise comparisons, we recognized six kinds of display behaviors, which depended on water turbidity and the number of speckles (Fig 2 and S2 Table), as follows:

1. 0% turbidity–few, a median number of, and many speckles. *T*. *melanogaster* showed a greater distance of visual fixation and approach and a lower latency compared with the other treatments (Fig 2; Table 3 and S2 Table). Orientation was greater than in the groups of snakes exposed to a turbidity higher than 20%. The percent of corrected classification was medium for this group of treatments (51%) and was more similar to snakes at 20% of turbidity exposed to fishes with many speckles (26%, S6 Table).
2. 20% turbidity–many speckles. The frequencies of slow crawling, orientation, and attack deployment in snakes toward fishes with more than 70 speckles in the pool were higher than toward fishes with few or a median number of speckles (Fig 2; Table 3 and S2 Table). The latency time was the lowest recorded in all of the analyzed groups (10.2 min). In this group, an increase in the number of speckles intensified the predatory behavior displayed by *T*. *melanogaster*. The percent of corrected classification was lower for this treatment (24%) and was more similar to snakes of group of treatments 5 (43%, S6 Table).
3. 20% turbidity–few and a median number of speckles. The deployment of predatory behavior in the snakes toward fishes with fewer than 70 speckles was characterized by an increase in approaches, with lower frequencies of slow crawling and attacks (S2 Table). The percent of corrected classification was medium for this group of treatments (38%) and was more similar to snakes at 20% of turbidity exposed to fishes with many speckles (22%, S6 Table). By contrast, the frequencies of orientations, latency, and the distance of visual fixation were near the mean (Fig 2 and Table 3).
4. 40% turbidity–many speckles. The deployment of predatory behavior in snakes toward fishes with more than 70 speckles was characterized by average values of the latency time, approaches, and visual fixation, but a higher frequency of attacks and slow crawling was observed compared with fishes with lower or intermediate numbers of speckles (Fig 2; Table 3 and S2 Table). This treatment do not differ statistically with 60% turbidity with few speckles, turbidity 40–60–80% with medium speckles, and 20% turbidity with many speckles. In this treatment, the increase in speckles intensified the depredatory behavior displayed by *T*. *melanogaster* with respect to 40% turbidity with few number speckles. However, this treatment showed the lowest percent of correct classification (5%, S6 Table).
5. 40% turbidity–few and a median number of speckles; and 60% turbidity–many, few, and a median number of speckles. The deployment of predatory behavior in snakes toward fishes in this group was characterized by decreases in attacks, slow crawling, orientation, approaches, and the distance of visual fixation compared with a turbidity below 60% (Fig 2; Table 3 and S2 Table). The number of speckles did not increase or reduce the depredatory behaviors. The percent of corrected classification was medium for this group of treatments (45%) and was more similar to group 6 of treatments (26%, S6 Table).
6. 80% and 100% turbidity–many, few, and a median number of speckles. The response of the snakes in a pool with high turbidity (80% and 100%) was statistically similar and independent of the number of speckles and was characterized by the greatest latency time and the lowest frequencies of orientation, slow crawling, approaches and attacks, and the shortest distance of visual fixation (Fig 2; Table 3 and S2 Table). The percent of corrected classification was high for this group of treatments (82%) and was more similar to group 5 of treatments (12%, S6 Table).

## Discussion

The results suggest that in a pool with high or zero turbidity, the number of lateral speckles on male *X*. *variata* does not appear to be a determining factor in the deployment of predatory behaviors of the snake *T*. *melanogaster*. Therefore, fish detection may occur through integration between chemical and visual signals of the fish, such as their body size, body shape, or speed [7,23], or based on another variable of the aquatic environment.

At 20% and 40% water turbidity, the predatory behavior of *T*. *melanogaster* toward male *X*. *variata* with a high number of lateral speckles increased, and visual fixation decreased according to the increase in water turbidity. Turbidity averaged nearly 60% in Lake Cuitzeo and nearly 20% at Lake Yuriria, and the male fish population from Lake Cuitzeo has fewer lateral speckles than that of Lake Yuriria. Under these conditions, it is predicted that in Lake Cuitzeo, the number of speckles on *X*. *variata* is not a factor that increases the frequency of predatory behavior in *T*. *melanogaster*, while in Lake Yuriria, it may increase the frequency of predatory behavior.

The number of lateral speckles on the fish in a pool with high turbidity could not be visualized efficiently by the snakes because the light in this water is dispersed due to the presence of suspended solids, and thus, the speckles are not visible due to the reflection of light. Therefore, the number of speckles on male *X*. *variata* could be irrelevant as an interspecific visual signal in environments with insufficient luminosity. Conversely, at low turbidity (20–40%), the number of lateral speckles on male *X*. *variata* imposes a higher predation risk due to attracting predators, such as the snake *T*. *melanogaster*.

Another component of the environmental context is the visual distance. Among aquatic specialist snakes, the minimum distance required for some *Thamnophis* species to achieve visual identification of prey is reported to be 2–6 cm [17]. In this study, the predatory behavior displayed by *T*. *melanogaster* in the pool reached a mean distance of 3.4 cm from the fish under all levels of water turbidity and for of the categories of speckles. This characteristic may be advantageous for aquatic snakes under low water turbidity; on the contrary, in high turbidity, the identification of potential prey could depend on other stimuli. The results showed that the latency time was longer when the snakes were exposed to higher water turbidity, and this time decreased when turbidity decreased. In the presence of predators, fish can reduce their movements or swim away to avoid predation [24]. Therefore, high water turbidity can have a negative effect on the time of predator foraging. This condition could occur in *T*. *melanogaster* when searching for prey in high turbidity aquatic systems. However, it is possible that *Thamnophis* snakes can compensate for low visibility in turbid aquatic environments by using the experience gained in foraging [25]. This leads us to consider the fact that *T*. *melanogaster* shows visual development that allows the detection of prey over considerable distances [26]. Vertebrate predators select conspicuous traits of prey visually and evaluate their vulnerability, but if the prey is hidden under high water turbidity, then the visual distance of aquatic snakes should be decreased [23].

The results of this study suggest that speckles on male *X*. *variata* are visual signals to potential predators such as the snake *T*. *melanogaster*. However, speckles are not a determining factor; snakes display a higher frequency of predatory behavior toward the fish, but their predatory behavior is influenced by water turbidity. Turbidity is considered a constraint that affects foraging in aquatic predators such as the snake *T*. *melanogaster* because it is involved in the visual identification of potential prey and capture success. Further experiments are needed to test the direct influence of snake predation on variation in sexual traits, such as the number of speckles on the flanks of male *X*. *variata*. Therefore, natural selection caused by predation may constrain the development of sexually dimorphic traits in *X*. *variata*, as suggested by other studies showing that male secondary sexual traits often increase the risk of predation [3,8,11].

## Supporting Information

S1 Fig Pictures of the three types of male fish *X*. *variata*. a) Male with few speckles (7–38 speckles). b) Median speckles (39–70). c) Many speckles (71–100). (TIF)

S1 Table Behavioral responses displayed by snake *T*. *melanogaster* over male fish *X*. *variata* with three speckles categories (few, median, and many) and water turbidity treatments (0–100%). (XLSX)

S2 Table Canonical scores, centroids, mean and standard deviation of first two roots obtained from discriminant function analysis from predatory behavior of snake *T*. *melanogaster* on male fish *X*. *variata*. The predatory behavior was registered under 18 treatments, depending of three categories of number of lateral iridescent specks (few, median, many) and water turbidity (100, 80, 60, 40, 20 and 0%).
(XLSX)

S3 Table Discriminant function coefficients to predict the treatment to which each observation belongs depending the behavioral response displayed by garter snake *T*. *melanogaster* over male fish *X*. *variata* with three speckles categories (few, median, and many) and water turbidity treatments (0–100%). (XLSX)

S4 Table Correct and incorrect classification of observations according to posterior probabilies from each treatment. The predatory behavior of *T*. *melanogaster* over male fish *X*. *variata* was registered under 18 treatments, depending of three categories of number of lateral iridescent specks (few, median, many) and water turbidity (100, 80, 60, 40, 20 and 0%).
(XLSX)

S5 Table Fisher values (superior diagonal) and probability (inferior diagonal) obtained from pairwise comparisons of treatments from discriminant function analysis. Values of the behavioral response of *T*. *melanogaster* on male fish *X*. *variata* with three speckles categories (few, median, and many) and water turbidity (0–100%).
(XLSX)

S6 Table Classification matrix of correct (diagonal) and incorrect (non-diagonal) observations according to posterior probabilities from each treatment. The predatory behavior of *T*. *melanogaster* over male fish *X*. *variata* was registered under 18 treatments, depending of three categories of number of lateral iridescent specks (few, median, many) and water turbidity (100, 80, 60, 40, 20 and 0%).
(XLSX)
