# Supplementary figures and images for: Visual Detection of Speckles in the Fish Xenotoca variata by the Predatory Snake Thamnophis melanogaster in Water of Different Turbidity
Source: PLoS One. 2015 Jun 10;10(6):e0129429. doi: 10.1371/journal.pone.0129429 (PMC4465328; doi:10.1371/journal.pone.0129429)

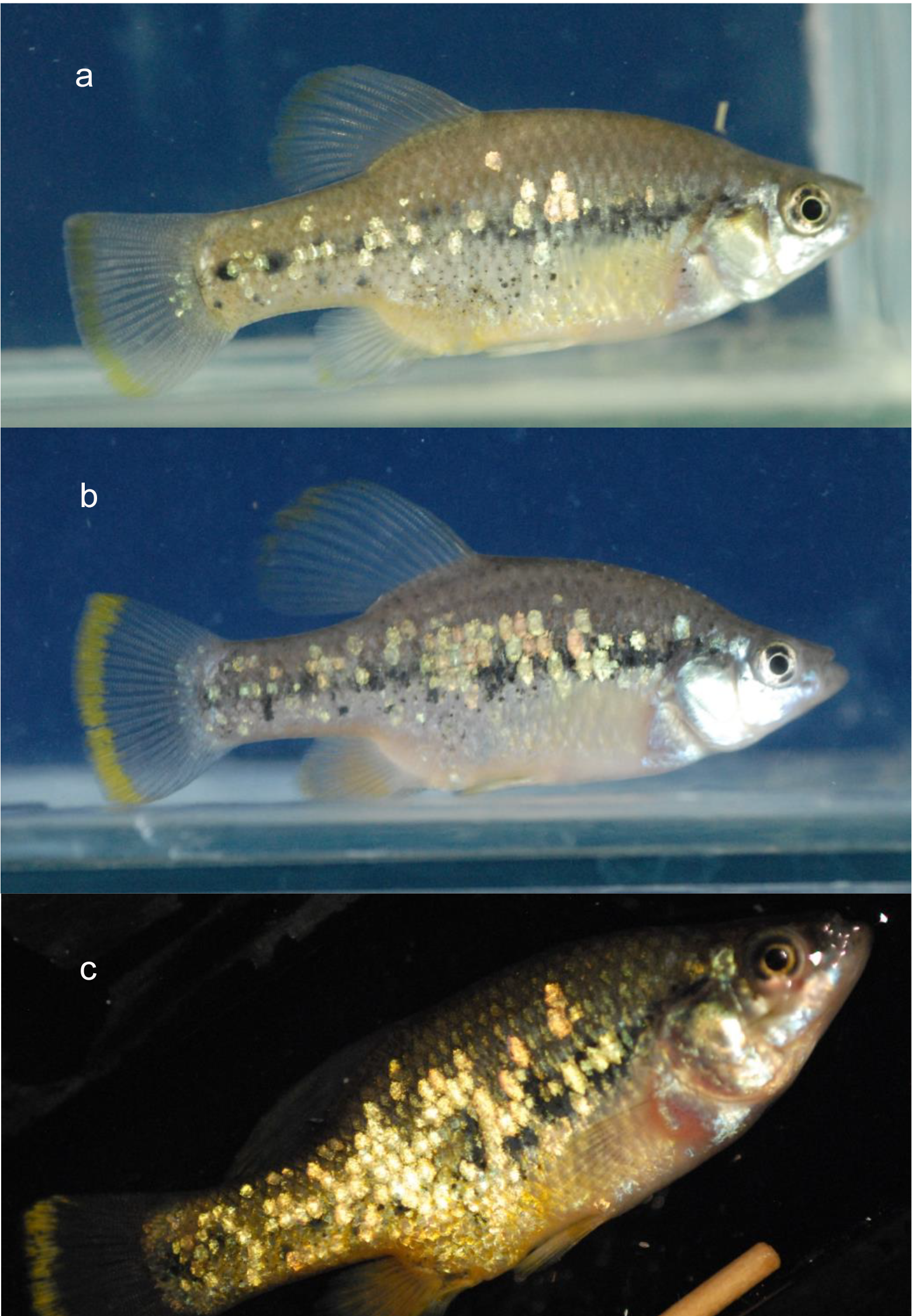

Supplement: S1 Fig — (TIF) [file pone.0129429.s001.tif]
